# Short-Term Efficacy and Safety of Scleral Lenses in the Management of Severe Dry Eye in a Chinese Population

**DOI:** 10.3390/jcm14030658

**Published:** 2025-01-21

**Authors:** Chuwei Lu, Danjie Han, Li Zeng, Jiaxu Hong, Daddi Fadel, Xingtao Zhou, Zhi Chen, Qihua Le

**Affiliations:** 1 Department of Ophthalmology, Eye, Ear, Nose & Throat Hospital of Fudan University, Shanghai 200031, China; lcw19858732824@163.com (C.L.); h2684078634@163.com (D.H.); doctzengli@163.com (L.Z.); jiaxu.hong@fdeent.org (J.H.); xingtaozhou@fudan.edu.cn (X.Z.); 2 NHC Key laboratory of Myopia and Related Eye Diseases, Shanghai 200031, China; 3 Shanghai Engineering Research Center of Synthetic Immunology, Shanghai 200032, China; 4 Department of Ophthalmology, Children’s Hospital of Fudan University, National Pediatric Medical Center of China, Shanghai 201102, China; 5 Centre for Ocular Research & Education, School of Optometry & Vision Science, University of Waterloo, Waterloo, ON N2L 3G1, Canada; daddi.fadel@uwaterloo.ca

**Keywords:** scleral lenses, dry eye, vision-related quality of life, efficacy, safety

## Abstract

**Background:** Scleral lenses (SLs) are recommended in DEWS II to treat dry eye (DE) patients that do not respond well to conventional therapies. This study aimed to evaluate the short-term (one month) efficacy and safety of SLs in the management of severe DE. **Methods:** This single-center prospective study enrolled 15 patients (22 eyes) who were diagnosed with severe DE. The Ocular Surface Disease Index (OSDI), the Chinese version of the 25-item National Eye Institute Visual Function Questionnaire (CHI-VFQ-25), and LogMAR best-corrected visual acuity (BCVA) were evaluated at baseline and one month following SL fitting. DE-related parameters were obtained and analyzed before and after one month of SL treatment, including tear-film breakup time (TBUT), corneal fluorescein staining (CFS), non-invasive breakup time (NIBUT), tear meniscus height (TMH), Schirmer I test (SIT), and meibomian gland (MG) dropout. Complications and adverse events were monitored. **Results:** OSDI scores (53.9 ± 28.1 vs. 10.4 (4.2–25), *p* = 0.0001) and CFS scores (10.2 ± 3.9 vs. 7 (0–12), *p* = 0.001) decreased after one month of SL therapy, while CHI-VFQ-25 scores (74.4 (54.8–83.8) vs. 95 (78.7–98), *p* = 0.0001) and TBUT (0.6 ± 0.5 vs. 2.2 ± 1.0, *p* < 0.0001) increased significantly. LogMAR BCVA improved from 0 (0–0.1) to 0 (0–0) (*p* = 0.0147). The average types of medications per eye decreased from 2.82 ± 1.01 to 1.32 ± 0.64 (*p =* 0.025), and the proportion of eyes using glucocorticoids significantly decreased from 63.6% to 13.6% (*p =* 0.001). No severe SL-related adverse events were reported. **Conclusions:** SL treatment quickly alleviated subjective symptoms as well as clinical signs of DE with good safety and enhanced the visual function and vision-related quality of life, showing its usefulness in the management of severe DE.

## 1. Introduction

Dry eye (DE), a chronic multifactorial ocular surface disease, is usually characterized by a loss of homeostasis of the tear film, ocular surface inflammation, tissue damage, and neurological abnormalities, and leads to ocular discomfort symptoms, visual impairment, and impaired quality of life [1,2,3]. Its prevalence in China ranges from 4.29 to 50.33% in different areas [4]. According to the classification of DE severity, eyes with corneal fluorescein staining (CFS) ≥ two quadrants, a tear-film breakup time (TBUT) < 2 s, or a Schirmer I test (SIT) score of 0 were classified as severe DE [3].

The management of severe DE is a clinical challenge. According to the four-tiered strategy put forward by TFOS DEWS II guidelines [5], routine treatments for DE, which were defined as Level 1 and 2 in the guidelines, were not enough for these cases. More importantly, ocular discomfort symptoms usually impair the visual quality of severe DE patients and their vision-related quality of life (VR-QoL) [6,7], making it necessary and critical that the treatments alleviate the symptoms rapidly.

Scleral lenses (SLs) are large-diameter, rigid, gas-permeable contact lenses that vault over the cornea and rest on the sclera. Unlike soft contact lenses, SLs can be customized to fit individual ocular anatomy with or without a toric design, thus creating a tear reservoir between the lens and cornea. For over a decade, SLs have been used to correct corneal irregularities caused by keratoconus or corneal surgeries [8,9]. In recent years, their application has been extended to the treatment of refractory DE, particularly in patients who fail to respond well to conventional therapies [10,11,12,13]. They are recommended in the management of moderate to severe DE cases (Level 3 in TFOS DEWS II), especially when the ocular surface is compromised [5].

However, currently, most studies mainly focus on its mid- to long-term effects, with an average follow-up ranging from 6 to 20 months [10,11,14]. Nevertheless, short-term efficacy in severe DE patients deserves attention because they usually experience severe ocular pain and need quick relief of symptoms. Moreover, the efficacy of SLs in Chinese patients has not been extensively explored, and they are more susceptible to developing DE than other races [15]. Additionally, consensus is still lacking regarding the indications and contradictions of SL treatment for DE patients, as well as standardized fitting protocols and complication management.

Therefore, we conducted this prospective cohort study in a Chinese population to investigate the one-month short-term efficacy and safety of SL therapy in the management of severe DE and its impact on visual quality and VR-QoL so as to provide clinical evidence for its application as a non-pharmacological alternative in the management of severe DE.

## 2. Materials and Methods

### 2.1. Study Design and Subjects

This single-center prospective study, conducted at the Eye, Ear, Nose & Throat Hospital of Fudan University, was executed in accordance with the Declaration of Helsinki and with the approval of the Ethics Committee of the hospital (2024016). Written informed consents were obtained from all participants.

A total of 15 patients who met the diagnostic criteria for severe DE according to “Chinese expert consensus on dry eye: definition and classification (2020)” [3] were recruited from September 2023 to May 2024. In brief, the patient should meet all of the following criteria in at least one eye: (1) TBUT < 2 s; (2) corneal epithelial damage ≥ two quadrants; (3) CFS ≥ 30 points, or presented as fused coarse dot/bulk staining, or with filamentary formations. Due to low repeatability and large variation, SIT was not included in the criteria, only except that when the SIT value was 0 mm/5 min, the subject was considered eligible even though the above three criteria were not all met.

The exclusion criteria were listed as follows: (1) ocular conditions affecting corneal sensation or integrity (e.g., herpetic keratitis, diabetic keratopathy); (2) severe conjunctival abnormalities that affected SL fitting (e.g., pterygium, conjunctivochalasis); (3) active ocular inflammation, infection, or diseases potentially impacting SL fitting (e.g., severe allergic conjunctivitis, uveitis, scleritis); (4) history of ocular surgery that might impair corneal sensation, such as corneal refractive surgery; (5) severe visually impairing disorders (e.g., glaucoma, vitreous hemorrhage, central corneal scar); (6) compromised immune response; (7) pregnancy, breastfeeding, or planning to conceive; (8) allergies to fluorescein sodium or inability to cooperate with lens fitting; (9) inability to comply with follow-up visits.

### 2.2. Ocular Examinations

#### 2.2.1. Order of Ocular Examinations

After the collection of demographic data including sex, age, concomitant systematic and ocular diseases, and the medications and DE therapies that had been used before planning treatment with SLs, all participants were asked to complete two validated questionnaires: the Ocular Surface Disease Index (OSDI) and a Chinese version of the 25-item National Eye Institute Visual Function Questionnaire (CHI-VFQ-25) [16,17]. The patients were given explanations if they could not understand the content of the questionnaires. All completed questionnaires were meticulously reviewed by the same researcher to confirm data completeness [6]. Then, each participant underwent the following examinations in sequence: best-corrected visual acuity (BCVA), slit-lamp biomicroscopy, anterior segment optical coherence tomography (AS-OCT), Oculus Pentacam, Corvis ST, Oculus Keratograph 5M (Oculus K5M), corneal sensitivity testing, and the Schirmer I test (SIT). TBUT and CFS were evaluated during slit-lamp biomicroscopy examination. All examinations were conducted by the same team composed of experienced ophthalmologists, optometrists, and trained technicians and were performed at the baseline and one month after regular SL wear.

#### 2.2.2. OSDI Questionnaire

The OSDI questionnaire, which consisted of 12 items covering ocular symptoms, vision-related functions, and environmental triggers, evaluated the severity of DE symptoms. Each item was scored from 0 (never) to 4 (always) or not applicable (N/A) based on the frequency of the patient’s experiences over the past week, and the final score ranged from 0 to 100, which was calculated with the following formula: (25 × total scores of all items answered)/number of questions answered. A higher score indicated more severe DE symptoms [18].

#### 2.2.3. CHI-VFQ-25 Questionnaire

The CHI-VFQ-25 questionnaire, which was validated [16] and used in previous studies [6,7], was applied to assess the VRQoL across 12 subscales: general health, general vision, near activity, distance activity, social function, color vision, peripheral vision, driving, role difficulties, ocular pain, dependency, and mental health. Each of the questions was scored from 0 (worst) to 100 (best), or N/A. The total scores were calculated following the guidelines provided by the National Eye Institute of the USA. A higher score equaled a better VR-QoL [19].

#### 2.2.4. BCVA

Snellen BCVA was measured and then converted to the logarithm of the minimal angle of resolution (LogMAR) format. The lower the LogMAR value, the better the visual acuity.

#### 2.2.5. Slit-Lamp Biomicroscopy

All patients underwent a slit-lamp examination to exclude any ocular abnormalities that could potentially affect SL fitting. TBUT and CFS were assessed under cobalt blue light, as previously described [6,7]. In brief, a sterile fluorescein strip (Jingmin, Tianjin, China), pre-moistened with sterile saline, was applied to the inferior conjunctival fornix. The participant was asked to blink 3–4 times naturally to ensure an even distribution of fluorescein on the ocular surface and to keep their eyes open as long as possible. The time from the last blink to the appearance of the first black spot or streak on the cornea was recorded as the TBUT. The test was performed three times. CFS was assessed within 3 min after fluorescein instillation. Based on the National Eye Institute grid [20], the cornea was divided into five parts: central, nasal, temporal, superior, and inferior. The fluorescence staining dots in each part were scored from 0 to 3 according to the following criteria: 0 for no staining; 1 for fewer than 15 dots; 2 for 16 to 30 dots; and 3 for more than 30 dots or strip/bulk staining or corneal filaments. The score for each part was added and the total ranged from 0 to 15.

#### 2.2.6. AS-OCT

AS-OCT (CASIA2, Tomey Corporation, Nagoya, Japan) was performed to obtain central corneal thickness (CCT) and central epithelial thickness (CET). During the examination, the subjects, in a seated position, had their heads stabilized with a chin and brow rest and were instructed to maintain a forward gaze. High-resolution images of the anterior eye segment were captured along the horizontal meridian in single-scan mode. The outcomes of CCT and CET were automatically output by the customized software. The thickness of the precorneal tear film during the SL fitting procedure was also evaluated by AS-OCT.

#### 2.2.7. Corneal Tomography

The corneal–scleral profile (CSP) mode of corneal tomography was performed to capture a series of CSPs using Pentacam (Oculus, Wetzlar, Germany), a high-precision non-contact Scheimpflug camera system. The participant was required to gaze forward, with superior, inferior, nasal, and temporal targets in a row to allow for the reconstruction of an 18 mm diameter corneal–scleral shape centered on the corneal vertex normal. The essential parameters such as sagittal height, horizontal visible iris diameter, and scleral angle were then derived for SL selection.

#### 2.2.8. Corvis ST

Corvis ST, a non-contact tonometer that assesses corneal deformation with high-speed Scheimpflug imagery, was used to measure biomechanically corrected intraocular pressure (bIOP). Patients, who were seated with their heads stabilized, were asked to hold their eyes open after several normal blinks. The measurement was automatically taken and repeated three times. The average value was calculated for analysis.

#### 2.2.9. Tear-Film Function

The Oculus K5M (Wetzlar, Germany) was used to evaluate the overall function of the tear film in a noncontact way. First, participants were told to blink twice in a dark environment and then to keep their eyes open until the system automatically recorded the first and average non-invasive breakup time (f-NIBUT, a-NIBUT). Then, the images of the lower tear meniscus were captured, and tear meniscus height (TMH) was measured using the built-in caliper. Finally, the upper and lower eyelids were gently everted to obtain images of the meibomian glands (MGs). The MG loss scores were assigned based on the proportion of dropout area: 0 for none, 1 for ≤1/3, 2 for 1/3 to 2/3, and 3 for >2/3. The lipid layer color (LLC) was qualitatively classified as normal (multicolor, red–green) or abnormal (blue–grey, hoary, achromatic), as previously reported [21].

#### 2.2.10. Corneal Sensitivity

The Cochet–Bonnet aesthesiometer (Luneau Ophthalmologie, Paris, France), featuring a filament with a diameter of 0.12 mm and a maximum length of 60 mm, was used for corneal sensitivity testing. Patients were seated and asked to look straight ahead. The central cornea was gently touched with the tip of the filament from the temporal side in a tangential direction. The test commenced at the maximal length (60 mm) and was decremented by 5 mm with each application until the patient reported a foreign body sensation on the cornea and the visible deflection of the filament by the examiner. This procedure was repeated three times, and the average value was taken for analysis.

#### 2.2.11. SIT

Without topical anesthesia, Schirmer paper strips (5 × 40 mm, Jingming, Tianjin, China) were carefully positioned into the outer one-third of the lower conjunctival sac cautiously to avoid irritative tearing. Patients were instructed to gently close their eyes for 5 min, after which the tear wetting length from the strip’s notch was measured and documented.

### 2.3. SL Fitting

SL fitting was conducted by one well-experienced ophthalmologist (ZC). Trial SLs were selected based on the parameters measured by Oculus Pentacam and AS-OCT. Before insertion, the SL was filled with non-buffered, non-preserved saline supplemented with fluorescein for enhanced visualization upon placement. When the SL was successfully settled, its position was immediately evaluated using slit-lamp biomicroscopy under white and cobalt blue light to ensure that its edge landed on the conjunctiva, neither contacting the cornea and the limbus nor exerting pressure on conjunctival vessels (Supplementary Figure S1). AS-OCT was then performed to evaluate the tear layer thickness beneath the lens, targeting a range of 200–300 µm for optimal moisture retention and minimal tear evaporation. These examinations were repeated 1 and 2 h after lens settlement for dynamic evaluation. Finally, trial SLs were removed, and rebound hyperemia or redness of the conjunctiva was checked [10,11]. Adjustments to the lens parameters, such as sagittal height, diameter, or edge design, were performed to achieve optimal fitting and ocular comfort. The fitting process was finalized with over-refraction with the SL in place to correct residual refractive errors and obtain the final prescription.

On the day of the SL delivery visit, the post-lens fluid reservoir and the lens alignment were evaluated again. After two hours of SL wear, the ocular surface was assessed after lens removal to check if any additional adjustments of lens parameters were needed to ensure the safety of lens wear. The patients were instructed to wear SLs for an average of 8–12 h per day, with overnight use explicitly avoided.

### 2.4. Statistical Analysis

Data at baseline and the one-month follow-up were analyzed using Stata 17.0 (StataCorp, College Station, TX, USA). The continuous data with normal distribution were described as mean ± standard deviation (SD) and analyzed with paired t-tests, otherwise they were presented as medians (P25–P75), and a Wilcoxon signed-rank test was performed. Categorical data were analyzed with Fisher’s exact test and the Chi-squared test. All statistical tests were conducted with a 95% confidence interval, and a two-tailed *p*-value < 0.05 was considered to indicate statistical significance.

## 3. Results

A total of 14 women and one man diagnosed with severe DE were enrolled in the study, with an average age of 48.3 ± 13.0 (14–73) years. The concomitant systematic diseases and ocular diseases related to severe DE included primary Sjögren’s syndrome (SS), graft-versus-host disease (GVHD), Stevens–Johnson syndrome (SJS), rheumatoid arthritis (RA), Vogt–Koyanagi–Harada (VKH) disease, thyroid eye disease (TED), and filamentary keratitis. After detailed examinations and careful evaluation, 22 eyes were prescribed SLs that were made of hexafocon A (13 eyes), Boston XO_2_ (eight eyes), and reflufocon D (one eye), respectively. The ranges of SAG, diameter (Dia), and base curves (BCs) were 3300~4500 um, 14.5~16.3 mm and 8.0~9.8 mm, respectively. The lenses were worn continuously for one month without overnight wear. The details of the demographic data and SLs are presented in Table 1.

### 3.1. Improvement of DE Symptoms and VR-QoL

**Figure 1:**
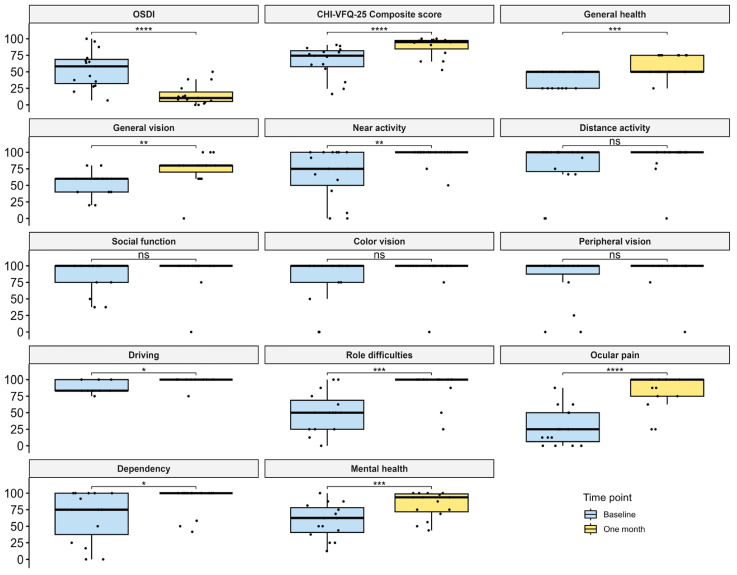
Comparison of OSDI scores, CHI-VFQ-25 composite scores, and CHI-VFQ-25 subscale scores between the baseline and after one month of SL therapy. Note: * *p* < 0.05; ** *p* < 0.01; *** *p* < 0.001; **** *p* < 0.0001; ns: no significance.

The OSDI score, CHI-VFQ-25 composite score, and scores for each subscale are presented in Figure 1. Compared to the baseline, a significant reduction in OSDI scores was found after one month of SL therapy (*p* = 0.0001). Meanwhile, the CHI-VFQ-25 composite scores significantly increased (*p* = 0.0001). Among the subscale scores of the CHI-VFQ-25, a notable improvement in the score for ocular pain was reported (median 28.3 vs. 100, *p* < 0.0001). Moreover, those for general health, general vision, near activity, driving, role difficulties, dependency, and mental health were also significantly increased (all *p* < 0.05).

### 3.2. Improvement of DE Signs

Compared to the baseline value, TBUT was remarkably prolonged after one month of SL therapy (*p* < 0.0001), along with reduced LogMAR BCVA (*p* = 0.0147) and decreased CFS scores (*p* = 0.001). Comparisons of the other parameters of DE did not show any statistical significance, except MG dropout scores, which were increased after one month (*p* = 0.039) (Table 2).

### 3.3. Dependency on Medication

The topical medications for DE were categorized into four types: artificial tears (with and without preservatives), serum extracts, immunosuppressants (e.g., cyclosporine), and glucocorticoids (e.g., fluorometholone). After one month of SL therapy, the average types of medications per eye significantly decreased from 2.82 ± 1.01 to 1.32 ± 0.64 (*p* = 0.025). Seventeen eyes (17/22, 77.3%) needed only one type of medication, and artificial tears were enough for 15 eyes (15/22, 68.2%) to control symptoms (Table 1). More importantly, the proportion of eyes using glucocorticoids dramatically decreased from 63.6% to 13.6% (*p* = 0.001).

### 3.4. Safety

During the one-month follow-up, one eye (4.6%) encountered subconjunctival hemorrhage with unknown etiology, which was spontaneously absorbed within 3 days. Additionally, 13 eyes (59.1%) reported mild midday fogging (MDF), which was resolved after removing, cleaning, and reapplying the lenses at noon or in the afternoon. It is notable that bIOP significantly decreased from 16.6 ± 3.3 mmHg to 15.3 ± 1.5 mmHg (*p* = 0.039). Importantly, neither severe adverse events such as microbial keratitis nor SL-induced conjunctival ischemia were found. After one month of SL therapy, corneal sensitivity, CCT, and CET did not have significant alterations compared to baseline values (Table 2).

## 4. Discussion

In the current study, remarkably decreased OSDI scores and CFS scores as well as improvement of TBUT were found after one month of SL therapy, indicating less ocular discomfort, better tear-film stability, and less corneal epithelial damage, which are crucial for ocular surface health. These findings revealed that SL treatment could alleviate the symptoms of ocular discomfort and improve clinical signs of severe DE within one month. SL wear not only kept ocular surface moisture stable and reduced tear evaporation [22] but also protected the ocular surface from environmental triggers such as smoke and dust during daily activities [23]. More importantly, SLs physically blocked direct contact between the eyelids and cornea, which avoided the shearing force from eyelids during blinking, thus facilitating corneal epithelial healing [24]. Notably, the patients were much less dependent on DE-related medications after SL therapy, especially the usage of corticosteroids, and benefited from decreased or discontinued use of glucocorticoids, as evidenced by decreased bIOP. All these findings supported that, as a non-pharmacological medical device, SL therapy might minimize the risks of long-term complications associated with chronic usage of various medications in the management of severe DE.

The current study is the first to report the impact of SLs on the VR-QoL of severe DE patients. Owing to fast epithelial recovery under the protection of SLs and the reestablishment of a smooth and regular optical interface [25,26], a great improvement in visual acuity was reported by patients after one month of treatment, along with a much better VR-QoL. It was notable that among all subscale scores, that for ocular pain presented the most significant improvement. Chronic ocular pain, which was quite common in severe DE patients, not only leading to anxiety [27,28] but also impairing their daily activities [6,29,30], was relieved by treatment with SLs, further contributing to improved general health and mental well-being.

The current study showed that one month of SL wear neither caused corneal edema nor affected corneal innervation. However, the incidence of MDF in our study was higher (59.1%) than that reported in previous studies (7%~46%) [31,32,33]. Abnormal alterations of lipid components (e.g., cholesteryl esters) and/or proportion (nonpolar lipid) were identified in eyes with MGD [34,35], which was one factor contributing to MDF [36]. Regarding the fact that the etiologies of severe DE, such as primary SS [37] and GVHD [38], were usually accompanied by MGD, the abnormalities of lipids in the tears of severe DE eyes made MDF more likely to occur during SL wear. Therefore, patient education on proper lens cleaning during the day is more crucial for these patients. Another unexpected safety issue finding was a slight increase in MG dropout scores after one month. Although one year of SL wear was reported to have no adverse effect on MG morphology in ametropic eyes [39], its impact on MGs in DE patients remained unclear. Given that MGD is quite common in severe DE eyes [37,38], it was reasonable to deduce that the MGs in these subjects were more fragile and more likely to be affected by the mechanical pressure exerted on eyelids by SLs. Further studies with longer follow-ups and more advanced MG imaging technology are needed to evaluate the long-term effect of SL wear on the structure and morphology of MGs in severe DE patients.

Several limitations should be acknowledged. First, the sample size is relatively small, and this is a single-center study, which might lead to selection bias. Second, the study was only performed in a Chinese population. It has been confirmed that compared to Caucasians, East Asians usually have smaller corneas, narrower limbi, tighter upper eyelids, and more congested anterior segments [40,41,42,43], making it difficult to compare the findings among different races. Third, the classification of DE, such as aqueous-deficient DE or evaporative DE, were not taken into consideration in the current study because the majority cases of severe DE, such as SS and GVHD, were mixed type, with both tear deficiency and over evaporation due to MGD being involved in the pathogenesis [37,38]. Further multicenter studies with larger sample sizes across ethnicities are needed to fully evaluate the efficacy and safety of SL therapy in the management of severe DE, and its long-term impacts on CCT, IOP, and MG morphology and function need more investigation.

## 5. Conclusions

In conclusion, SL therapy alleviates the symptoms and signs of severe DE within one month by providing continuous hydration and mechanical protection for the ocular surface. Meanwhile, it significantly improves the visual function and VR-QoL. No severe adverse events were found during one month of usage. SLs may serve as a potential alternative treatment for patients with refractory DE. Their long-term efficacy, safety, and potential use in various ethnic populations need further investigation.

## Figures and Tables

**Table 1:** Demographic data and SL parameters of all participants.

	Gender	Age	Concomitant Systematic and Ocular Diseases	Previous Ocular Surface Surgeries	Eyes	Physical Treatment and Medications Before SL Therapy	Medications After SL Therapy	BCVA	Lens Materials	SAG (um)	Dia (mm)	BC (mm)	CT (mm)	BVP (D)	Cyl (D)	Axis (°)	Adverse Events	
																	Midday Fogging	Others
Patient 1	Female	38	SS	No	OD	IPL; Preserved AT (3); Preservative-free AT (1); Cyclosporine; Loteprednol	Preservative-free AT (1)	0	hexafocon A	3900	16.00	8.05	0.30	−5.25	−0.75	80	Yes	No
					OS	Same as OD	Same as OD	0	hexafocon A	3900	16.00	8.05	0.30	−3.75	−0.50	100	Yes	No
Patient 2	Female	46	SS	No	OD	Preserved AT (1); Serum extracts; Cyclosporine; Fluorometholone	Preserved AT (1);	0	hexafocon A	3700	15.60	8.25	0.30	+3.50	−0.25	80	No	No
					OS	Same as OD	Preservative-free AT (1)	0	hexafocon A	3700	15.60	8.25	0.34	+5.75	−0.25	100	No	No
Patient 3	Female	59	TED	No	OS	Preserved AT (1); Serum extracts; Cyclosporine	Preserved AT (1); Cyclosporine	0	hexafocon A	3400	14.80	8.45	0.30	+3.00	−0.25	0	Yes	No
Patient 4	Female	58	No	Excision of pinguecula	OD	Preserved AT (1); Fluorometholone; Cyclosporine	Preservative-free AT (1); Cyclosporine	0.22	Boston XO_2_	3300	14.50	9.10	0.42	+1.00	−0.36	180	No	No
Patient 5	Female	51	GVHD	No	OD	IPL; Preserved AT (1); Serum extracts; Fluorometholone	Preservative-free AT (1)	0.05	Boston XO_2_	3500	14.50	8.20	0.42	+3.00	−0.36	180	Yes	No
Patient 6	Female	46	SS	Amniotic membrane transplantation	OD	Preserved AT (2); Preservative-free AT (1); Serum extracts; Loteprednol; Fluorometholone	Preservative-free AT (1)	0	Boston XO_2_	3400	14.50	8.60	0.42	0.50	−0.36	180	Yes	Subconjunctival hemorrhage of undetermined etiology
Patient 7	Female	47	SS	No	OD	IPL; Preserved AT (1); Loteprednol	Preserved AT (1)	0	Boston XO_2_	3600	14.50	8.42	0.42	1.25	−0.36	180	No	No
Patient 8	Female	44	SJS	No	OS	Preserved AT (2); Serum extracts; Cyclosporine; Tacrolimus; Fluorometholone	Preservative-free AT (1); Fluorometholone; Cyclosporine	0.70	hexafocon A	3700	15.60	8.25	0.30	+1.25	−1.00	105	Yes	No
Patient 9	Female	54	RA	No	OD	IPL; Punctal Occlusion; Preserved AT (1); Preservative-free AT (1); Cyclosporine	Preserved AT (1)	0	Boston XO_2_	3400	14.50	8.60	0.30	−2.50	−0.36	180	No	No
					OS	Same as OD	Same as OD	0.05	Boston XO_2_	3400	14.50	8.60	0.30	−1.00	−0.36	180	No	No
Patient 10	Female	57	No	No	OD	Preserved AT (1)	Preservative-free AT (1)	0	hexafocon A	3900	15.60	8.05	0.30	+3.50	−0.25	0	Yes	No
					OS	Same as OD	Same as OD	0	hexafocon A	3900	15.60	8.05	0.30	+4.25	−0.50	90	Yes	No
Patient 11	Female	45	VKH	No	OD	IPL; Preserved AT (1); Preservative-free AT (1); Serum extracts; Fluorometholone	Preservative-free AT (1); Fluorometholone	0	Boston XO_2_	3900	16.00	9.80	0.42	5.00	−0.36	180	Yes	No
					OS	Same as OD	Preservative-free AT (1)	0	Boston XO_2_	3.90	16.00	9.80	0.42	6.75	−0.36	180	Yes	No
Patient 12	Female	73	SS	No	OD	Preserved AT (1) Serum extracts	Preserved AT (1); Preservative-free AT (1)	0.10	hexafocon A	3400	15.20	8.45	0.31	+4.75	−0.25	0	Yes	No
					OS	Same as OD	Same as OD	0.10	hexafocon A	3400	15.20	8.45	0.33	+5.50	−0.25	0	Yes	No
Patient 13	Female	14	Filamentary keratitis	No	OD	Preservative-free AT (2); Serum extracts; Cyclosporine; Loteprednol; Fluorometholone	Cyclosporine	0	hexafocon A	4000	16.30	8.25	0.30	−1.00	−0.25	0	No	No
					OS	Preservative-free AT (2); Serum extracts; Tacrolimus; Loteprednol; Fluorometholone	Tacrolimus	0	hexafocon A	4000	16.30	8.25	0.30	−2.00	−0.50	100	No	No
Patient 14	Male	39	GVHD	No	OD	Preserved AT (1); Tacrolimus; Fluorometholone	Preserved AT (1); Tacrolimus; Fluorometholone	0	hexafocon A	3700	15.60	8.25	0.30	+1.75	−1.50	90	No	No
Patient 15	Female	54	No	No	OD	Serum extracts	Preservative-free AT (1)	0.22	reflufocon D	4510	15.80	8.04	0.31	2.00	−1.50	145	Yes	No

Abbreviations: SAG, Sagittal depth; Dia, diameter; BC, base curve; CT, center thickness; BVP, back vertex power; Cyl, cylinder; AT, artificial tears; IPL, intense pulsed light; SS, Sjögren’s syndrome; VKH, Vogt–Koyanagi–Harada disease; TED, thyroid eye disease; GVHD, graft-versus-host disease; SJS, Stevens–Johnson syndrome; RA, rheumatoid arthritis; BCVA, best corrected visual acuity. Note “Preservative-free AT” refers to AT that do not contain preservatives, while “Preserved AT” refers to AT containing preservatives. The number in parentheses following “AT” indicates the number of AT types that the patient used at that time.

**Table 2:** Comparisons of the clinical signs and DE parameters between the baseline and after one month of SL treatment.

	Baseline	One Month	*p*
LogMAR BCVA	0 (0–0.1)	0 (0–0)	0.015 *
ST (s)	2.5 (0–9)	2 (0–5)	0.626
TBUT	0.6 ± 0.5	2.2 ± 1.0	<0.0001 *
CFS	10.2 ± 3.9	7 (0–12)	0.001 *
First NIBUT	3.7 (2.9–5.7)	6.1 ± 3.3	0.109
Average NIBUT	5.1 (3.4–7.6)	8.1 ± 3.9	0.282
TMH	0.2 (0.2–0.3)	0.2 ± 0.1	0.445
MG dropout scores	2 (1–3)	2.7 ± 1.6	0.039 *
LLC			0.761
Normal	10 (45.5%)	9 (40.9%)	
Abnormal	12 (54.5%)	13 (59.1%)	
Corneal sensitivity	31.6 ± 23.9	39.6 ± 20.0	0.078
CCT	517.1 ± 49.9	526.7 ± 52.6	0.055
CET	53.4 (50.3–55.6)	53.5 (50.2–56.1)	0.429
bIOP	16.6 ± 3.3	15.3 ± 1.5	0.039 *

* means a *p*-value less than 0.05. Abbreviations: ST, Schirmer test; TBUT, tear-film breakup time; CFS, corneal fluorescein staining; CCT, central corneal thickness; CET, corneal epithelial thickness; NIBUT, non-invasive break-up time; TMH, tear meniscus height, MG, meibomian gland; LLC, lipid layer color; bIOP, biometric intraocular pressure.

## Data Availability

Data are contained within the article/Supplementary Material.

## Supplementary Materials

The following supporting information can be downloaded at https://www.mdpi.com/article/10.3390/jcm14030658/s1, Figure S1: Assessment of SL fitting under slit-lamp biomicroscopy.
