# Supplementary material for: Short-Term Efficacy and Safety of Scleral Lenses in the Management of Severe Dry Eye in a Chinese Population
Source: J Clin Med. 2025 Jan 21;14(3):658. doi: 10.3390/jcm14030658 (PMC11818766; doi:10.3390/jcm14030658)
Supplement: Supplementary file 1 [file jcm-14-00658-s001.zip › jcm-3333919-supplementary.pdf]

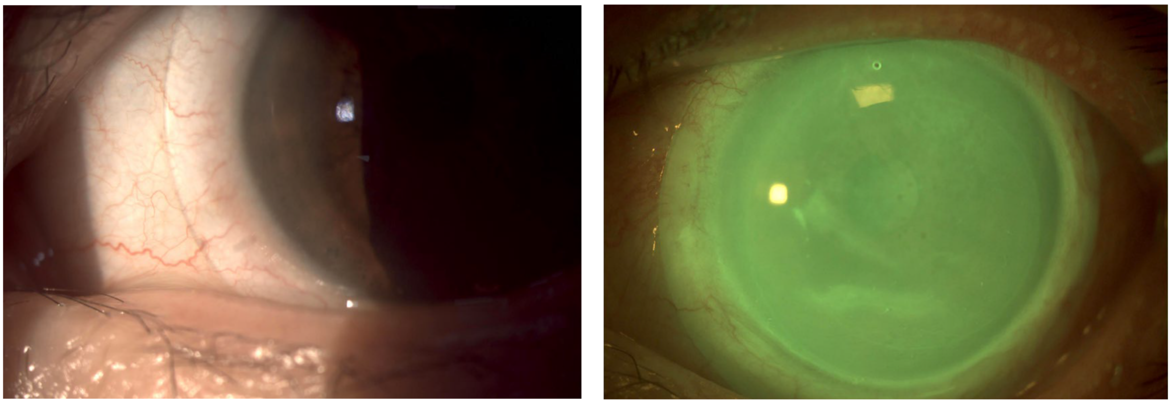

a b

Figure S1. Assessment of SL fitting under slit-lamp biomicroscopy. Under white light, the SL edge did not cause any signs of conjunctival compression or present edge lift. The vessels remained open and unobstructed **(a)**. Under cobalt blue light, the SL was shown to land on the conjunctiva without contacting the cornea or limbus **(b)**.
